# Effect of Graded Facetectomy on Lumbar Biomechanics

**DOI:** 10.1155/2017/7981513

**Published:** 2017-02-19

**Authors:** Zhi-li Zeng, Rui Zhu, Yang-chun Wu, Wei Zuo, Yan Yu, Jian-jie Wang, Li-ming Cheng

**Affiliations:** ^1^Spine Division of Orthopaedic Department, Tongji Hospital, Tongji University School of Medicine, 389 Xincun Road, Shanghai 200065, China; ^2^Department of Histology and Embryology, Tongji University School of Medicine, 1239 Siping Road, Shanghai 200092, China

## Abstract

Facetectomy is an important intervention for spinal stenosis but may lead to spinal instability. Biomechanical knowledge for facetectomy can be beneficial when deciding whether fusion is necessary. Therefore, the aim of this study was to investigate the biomechanical effect of different grades of facetectomy. A three-dimensional nonlinear finite element model of L3–L5 was constructed. The mobility of the model and the intradiscal pressure (IDP) of L4-L5 for standing were inside the data from the literature. The effect of graded facetectomy on intervertebral rotation, IDP, facet joint forces, and maximum von Mises equivalent stresses in the annuli was analyzed under flexion, extension, left/right lateral bending, and left/right axial rotation. Compared with the intact model, under extension, unilateral facetectomy increased the range of intervertebral rotation (IVR) by 11.7% and IDP by 10.7%, while the bilateral facetectomy increased IVR by 40.7% and IDP by 23.6%. Under axial rotation, the unilateral facetectomy and the bilateral facetectomy increased the IVR by 101.3% and 354.3%, respectively, when turned to the right and by 1.1% and 265.3%, respectively, when turned to the left. The results conclude that, after unilateral and bilateral facetectomy, care must be taken when placing the spine into extension and axial rotation posture from the biomechanical point of view.

## 1. Introduction

Lumbar stenosis is one of the leading sources of lower back pain worldwide. It is defined as a narrowing of the lumbar spinal canal [1] due to degeneration of the spinal canal and neural foramen. An estimated 73 million people will be over the age of 65 of which 30% are projected to have symptomatic lumbar spinal stenosis in the US by the year 2030 [1]. Surgery is typically required for patients with lumbar stenosis over the age of 65 years [2]. Although nonoperative treatments with accompanying lifestyle modifications and disc microsurgery are becoming more and more popular, the gold standard treatment for lumbar stenosis is still open surgery [2]. The most common surgery for decompression is facetectomy and laminectomy, with the choice of unilateral or bilateral intervention depending on the degree of stenosis. An unfortunate but unavoidable downside to removing anatomical structures of the spine is an altered load-bearing and motion environment. Greater spinal instability and larger deformation may occur. Knowing the level of instability under physiological loading can help the surgeon to decide whether additional spinal fusion is necessary.

Several groups have reported on the biomechanical behavior of the spine after resecting dorsal lumbar regions using in vitro experimental studies. In 1990, Abumi et al. [3] showed that removing supraspinous/interspinous ligaments did not affect the range of motion but that total facetectomy made the spine unstable. Okawa et al. [4] applied cyclic compressive and bending loads to a cadaveric spinal unit simulating partial facetectomy with intact spinous processes and ligaments. The results showed that facetectomy did not have a significant effect on flexion, but there was a significant effect on compression and extension. Similarly, Zhou et al. [5] performed in vitro unilateral graded facetectomy on 5 cadavers and failed to find any significant negative effects to the range of flexion and extension. However, if the range of graded facetectomy exceeded 50%, spinal stability under lateral bending and axial rotation was greatly impacted. Saying that, the use of cadaveric experiments presents several limitations. The number of the cadaveric specimens is limited and the individual differences in anatomy are not reproducible across multiple experiments. Also, most specimens are from elderly individuals with variations in bone quality [6].

Finite element (FE) analysis is an important method for biomechanical investigations. Material properties can be varied and geometries can be generated and manipulated as desired according to different aims of studies. A number of FE studies around facetectomy have been reported in the literature. In 2003, Zander et al. [7] used a validated FE model to study both facetectomy and laminectomy, recording parameters such as motion, intradiscal pressures (IDP), stress, and facet joint forces. However, only standing and forward bending were investigated. Lee and Teo [8] investigated different spinal motions after laminectomy using a L2-L3 lumbar FE model. The results showed that total laminectomy increased motion and annulus stress, except when under lateral bending. Chen et al. [9] found that posterolateral fusion with hemilaminectomy may relax the stress concentrations on the intervertebral disc above the fusion mass when placed in flexion. Kiapour et al. [10] evaluated the biomechanical mechanism of Dynesys dynamic stabilization which was a semirigid pedicle screw fixation system for graded facetectomy. More recently, Erbulut [11] created an asymmetric FE model of the lumbar spine and subjected it to graded facet injuries in order to study the effect on the range of motion. Total left unilateral medial facetectomy, total bilateral facetectomy, 50% unilateral medial facetectomy, and 75% unilateral medial facetectomy were modeled. However, only the medial section of the segment was involved and only motion parameters were calculated. Involving more parameters, such as pressure and stress, under a range of spinal postures would help to create a more comprehensive biomechanical understanding of the environment in the spine after graded facetectomy.

Therefore, the aim of this study is to construct an FE model of a spinal segment and investigate the biomechanical effect of graded facetectomy on intervertebral rotation (IVR), intradiscal pressure, facet joint forces, and maximum von Mises equivalent stresses for flexion, extension, left/right lateral bending, and left/right axial rotation.

## 2. Materials and Methods

### 2.1. Finite Element Model of L3–L5

**Figure 1:**
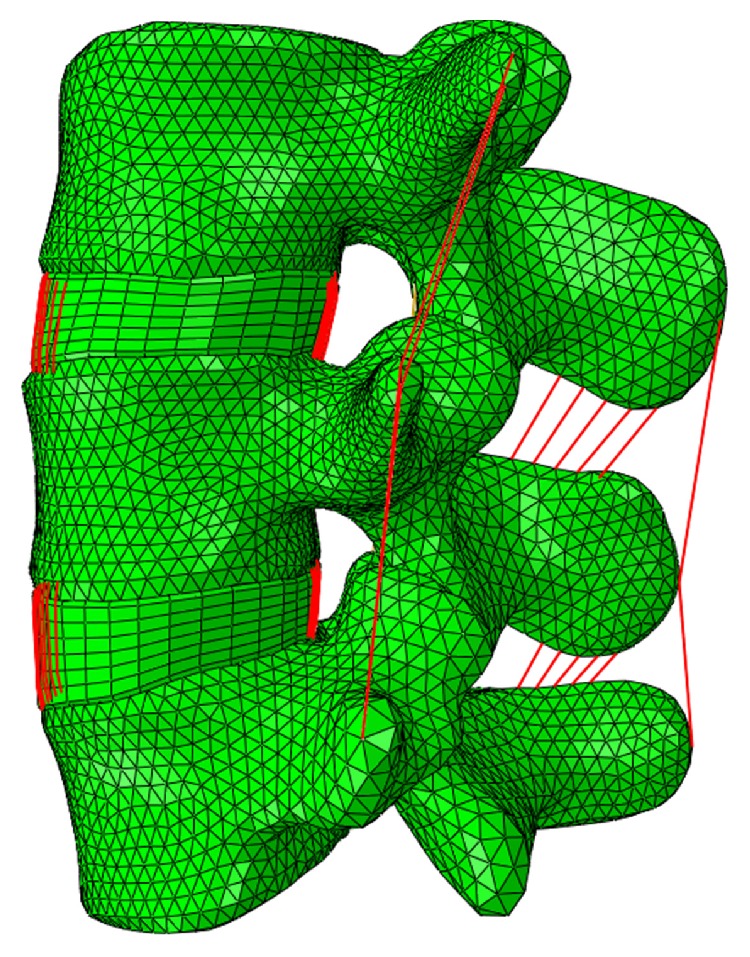
Finite element model of L3–L5.

A nonlinear finite element model of L3–L5 was constructed from CT image data obtained from a 25-year-old Chinese male without any history of spinal disease. The CT images saved as Digital Imaging and Communications in Medicine format were imported into Simpleware Software (Simpleware Ltd.). After segmentation, feature extraction, smoothing, and mesh processes, the elements and nodes were imported to an FE software for remesh. The vertebrae were meshed using tetrahedral elements, and the intervertebral discs were meshed using hexahedral elements in the ABAQUS software. The FE model consisted of 32,850 nodes and 96,970 elements. Each vertebra consisted of a cortical shell, a cancellous core, and a posterior bony structure. The 0.5 mm thick cortical shell [12] and the posterior bony structure were modeled as isotropic elastic materials, while the cancellous core was modeled as transverse isotropic. The cartilaginous endplates were 0.8 mm thick [13]. Each intervertebral disc was composed of an incompressible nucleus pulposus and surrounding annulus fibrosus. Rebar elements of two times seven layers were used to represent the fiber and the fiber stiffness decreased from the outside towards the centre [14]. The vertebrae and intervertebral discs were tied together. There was a gap of 0.5 mm [15] between the curved facet joints, and a thin cartilaginous layer of 0.25 mm was created for each facet articular surface. All seven ligaments of the lumbar spine were integrated according to their anatomical positions and were represented by tension-only spring elements with nonlinear material properties [16]. The FE model of L3–L5 is shown in Figure 1, and the material properties are shown in Table 1.

### 2.2. Validation

To validate the model, a moment of 7.5 Nm was applied to the top surface of L3 in the direction of flexion, extension, right lateral bending, and right axial rotation. The inferior endplate of L5 was rigidly fixed. The IVR of L4-L5, the region of concern in this study, was calculated and compared with in vitro data [17]. In addition, the IVR of L3–L5 was compared with in vitro data from whole lumbar specimens [18]. As a whole lumbar spine has five vertebrae and four spinal motion units, a direct comparison is unsuitable. Therefore, a ratio for IVR between L3–L5 and L1–L5 was adopted according to data from Pearcy et al. [19, 20]. This ratio was calculated for flexion-extension, lateral bending, and axial rotation, and the IVR of L3–L5 was justified according to this ratio. A subsequent 500 N axial compressive follower load was also applied and the IDP was estimated and compared with in vivo data [28].

### 2.3. Graded Facetectomy Model

Starting from the intact model, different graded facetectomies were simulated by modifying the facet joint of L4-L5 with the facet capsular ligament: 50% unilateral facetectomy, total left unilateral facetectomy, and total bilateral facetectomy. Regarding 50% unilateral facetectomy, different portions could be removed, depending on the surgical approaches. Therefore, to study sensitivity, four different 50% unilateral facetectomies were simulated by removing the upper, lower, outer, and medial portions of the left facet joint of L4-L5, respectively.

### 2.4. Boundary and Loading Conditions

The inferior endplate of L5 was rigidly fixed as a boundary condition. Flexion, extension, right lateral bending, left lateral bending, right axial rotation, and left axial rotation of the upper body were investigated. All loads (Table 2) were chosen according to Rohlmann et al. [23, 24] and Dreischarf et al. [25, 26]. The finite element program ABAQUS, version 6.13 (Dassault Systèmes, Versailles, France) was used for the simulations.

## 3. Results

### 3.1. Validation

**Figure 2:**
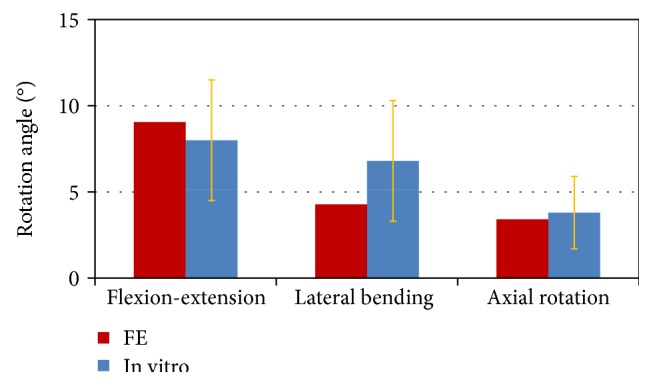
Comparison of the calculated intervertebral rotations of L4-L5 in the finite element (FE) model against experimental data [17] under a moment of 7.5 Nm for different loading cases.

**Figure 3:**
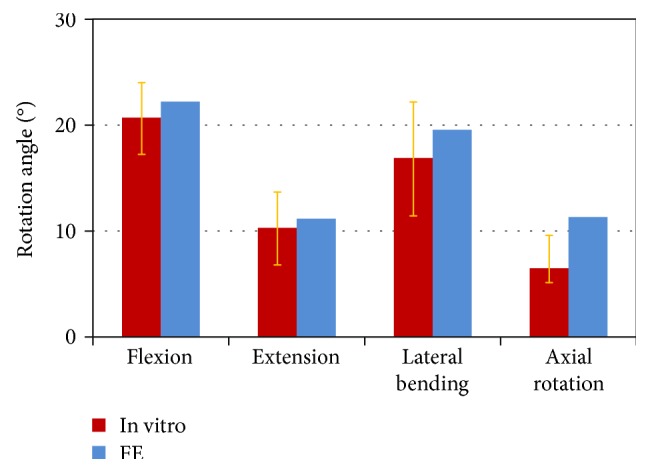
Comparison of the rotations in the finite element (FE) model and measured (Rohlmann et al. [18]) rotations in the lumbar spine under a moment of 7.5 Nm for different loading cases.

The calculated IVR of the L4-L5 motion segment was within the range of in vitro experimental data [17] (Figure 2). Regarding the overall rotation, the estimated IVR was compared with in vitro data (Figure 3). The mobility of the model in flexion-extension and lateral bending was inside the range measured for seven lumbar specimens [18]. The mobility of the model in axial rotation was slightly outside, but the mobility for a single motion segment was still within the range of other published data [27]. Regarding the axial compressive load, the estimated IDP of L4-L5 in a standing position was 0.44 MPa. This is comparable to in vivo measurements by Wilke et al. [28], who recorded 0.50 MPa for spinal loading.

### 3.2. Intervertebral Rotation

**Figure 4:**
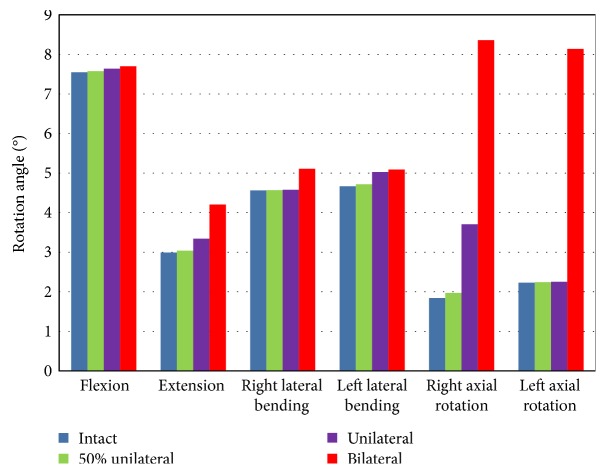
The values of rotation angles in each motion plane for the intact model and graded facetectomy models.

The rotation angles in each motion plane for the intact model and graded facetectomy models are summarized in Figure 4. The values presented for the 50% unilateral facetectomy were the mean values calculated for the four different 50% unilateral facetectomies simulated. In flexion, graded facetectomy had only a minor effect. In extension, unilateral facetectomy increased IVR by 11.7% and bilateral facetectomy increased IVR by 40.7%. For right lateral bending, unilateral facetectomy and bilateral facetectomy increased the IVR by 0.3% and 11.9%, respectively, while for left lateral bending, this was 7.7% and 9.0%, respectively. In general, facetectomy had a large effect on the axial rotation. The 50% unilateral facetectomy, unilateral facetectomy, and the bilateral facetectomy increased the right axial rotation of the L4-L5 motion segment by 7.2%, 101.3%, and 354.3%, respectively, and by 0.6%, 1.1%, and 265.3%, respectively, for left axial rotation. For all loading types, the 50% facetectomy only increased the IVR by a maximum of 7.2%, which occurred under right axial rotation.

In most times, different types of partial resection resulted in similar IVR with a difference of less than 2%. Only for right axial rotation, removing the lower and outer portion of the left L4-5 facet joint increased the IVR by 6.4% (0.12°) and 19.2% (0.35°), respectively.

### 3.3. Intradiscal Pressure and Facet Joint Force

In most cases, facetectomy had only a minor influence on the IDP. In extension, the unilateral facetectomy and the bilateral facetectomy increased the IDP by 10.7% and 23.6%, respectively, and for left axial rotation, the bilateral facetectomy increased the IDP by 9.6%. The extension movement also produced the greatest facet joint force on the contralateral facet joint. For this loading case, the 50% unilateral facetectomy increased the contralateral facet joint force by 25% on average and the total unilateral facetectomy increased the force by 108.1%.

### 3.4. Maximum von Mises Equivalent Stresses in the Annuli

The four 50% unilateral facetectomy procedures only resulted in slightly different results for maximum von Mises stress in the annuli in comparison to the intact model. Unilateral facetectomy increased the maximum von Mises stress in the annuli by 13.1% in extension and 23.5% in right axial rotation. Bilateral facetectomy increased the maximum von Mises stress in the annuli by 32.3% in extension and 59.3% in axial rotation.

## 4. Discussion

An FE model of L3–L5 was constructed in this study, and the mobility of the model and the IDP were calculated for validation study. The effect of graded facetectomy on intervertebral rotations, intradiscal pressure, facet joint forces, and maximum von Mises equivalent stresses in the annuli was analyzed for all six loading conditions.

Regarding validation for the overall rotation, the calculated overall IVR of L3–L5 was justified. For example, the calculated axial rotation of L3–L5 was 6.54° in our FE model under a 7.5 Nm moment. According to Pearcy et al., the ratio between the rotation of L3–L5 and L1–L5 is 60% [19, 20]. The estimated axial rotation of L1–L5 in our model was 10.9° (6.54°/60%). Figure 3 showed the comparison between the justified IVR of L1–L5 and in vitro data [18]. All calculated data for the IDP, the IVR of L4-L5, and the overall IVR were inside the range of in vivo/in vitro data, respectively. Therefore, the load and mobility of the FE model were in the physiological range.

For the four 50% unilateral facetectomies simulated, there were no significant differences in results after resecting different portions of the vertebra compared with the intact model. Fusion or dynamic stabilization may not be necessary. Similarly, Zhou et al. [5] also concluded that lumbar stability was not significantly affected if graded facetectomy was performed to remove less than 50% of bone, which is the same as our finding. In right axial rotation, removing the lower and outer portions of the left L4-5 facet joint increased the IVR by 6.4% and 19.2%, respectively. Although the absolute values were only 0.12° and 0.35°, retaining these portions of the bone may be beneficial. Choi et al. [29] also suggested that the resection should not involve the articular surface as preserving a larger articular surface is important for maintaining spinal stability.

This study demonstrated that total unilateral and bilateral facetectomy had little impact on the IVR in flexion and lateral bending, which is similar to in vitro results reported by Quint et al. [30]. These two facetectomy procedures also had a minor influence on the IDP and facet joint forces in flexion and lateral bending. For extension, total unilateral and bilateral facetectomy increased the IVR by 11.7% and 40.7%. After total unilateral facetectomy, the contralateral facet joint force increased by 108.1% in extension. This was the largest increase in contralateral facet joint force among all loading cases. At the same time, the increased IDP and the maximum von Mises stresses in the annuli in this model indicated a greater load through the intervertebral disc of L4-L5. This would inevitably lead to a greater risk of intervertebral disc degeneration and arthritis of the facet joints. Therefore, extension postures need to be achieved with care after total unilateral/bilateral facetectomy.

The facetectomy had a significant effect on IVR in axial rotation. Notably, after bilateral facetectomy, the IVR for right and left axial rotation increased by 354.3% and 265.3%. This is comparable to results from the literature [11]. Besides spinal instability, greater IDP of L4-L5 intervertebral disc and stress in the annuli will result in rupture of the annulus fibrosus. These remind us that axial rotation is another position needed to be treated carefully from the biomechanical point of view. Meanwhile, due to the change of stability, fusion or dynamic stabilization would be needed to reconstruct the lumbar stability from the biomechanical results. In this study, only the biomechanical aspects were regarded, but clinical experiences are inevitable as well; a cooperation of surgeons and bioengineers could induce the individual optimum for specific patients.

The majority of previous publications on spinal biomechanics constructed models based on the anatomy of European or American subjects [7, 10, 11]. However, differences in anatomy have been demonstrated between European or American people and Asian people, especially for the orientation of the facet joint. Grogan et al. [31] reported a mean facet joint angle of the lumbar spine from American subjects to be 37°. Yang and Wang reported this to be 47° for the lumbar spine of Chinese [32], while the value for Thais measured by Pichaisak et al. was 46° [33]. Given the vast and ever-growing Asian population, a concise FE analysis of graded facetectomy may be of great benefit across an array of disciplines and professions for Asians.

Some limitations of this study should be noted. In the case of spine, only a few parameters such as intervertebral rotation and intradiscal pressure were measurable and thus suitable for validation. Therefore, facet joint forces and the maximum von Mises equivalent stresses in the annuli were presented only by relative ratios. The model used for simulation was from an asymptomatic volunteer. The effects of different pathological factors on the stability of spine after graded facetectomy need to be investigated in further study. In addition, the effects of muscle forces and bone mineral density on facetectomy need further study as well. Although several simplifications were made, the reported results are reliable because the same parameters were chosen for all loading cases. The results in the present study should be viewed as a comparative analysis between graded facetectomy models and an intact model for all six spinal loading conditions.

## 5. Conclusions

The results conclude that, after unilateral and bilateral facetectomy, care must be taken when placing the spine into extension and axial rotation posture from the biomechanical point of view.

## Figures and Tables

**Table 1:** Material properties used for the different tissues in the finite element model.

Component	Elastic modulus (MPa)	Poisson ratio	References
Cortical bone	10,000	0.30	[16]
Cancellous bone (transverse isotropic)	200/140 (axial/radial)	0.45/0.315	[21]
Posterior bony structures	3,500	0.25	[14]
Ligaments	Nonlinear		[16]
Cartilage of endplate	Hyperelastic, neo-Hookean, *C*_10_ = 0.3448, *D*_1_ = 0.3	
Nucleus pulposus	Incompressible		[16]
Ground substance of annulus fibrosis	Hyperelastic, neo-Hookean, *C*_10_ = 0.3448, *D*_1_ = 0.3		[22]
Fibers of annulus fibrosis	Stiffness decreased from the outer to the centre		[14]
Facet joint	Soft contact		[15]

**Table 2:** Loads used to simulate flexion, extension, lateral bending, and axial rotation.

	Flexion	Extension	Lateral bending	Axial rotation
Rohlmann et al. [23, 24]	1175 N + 7.5 Nm	500 N + 7.5 Nm	—	—
Dreischarf et al. [25, 26]	—	—	700 N + 7.8 Nm	720 N + 5.5 Nm
